# Biological Invasion of Fish Parasite *Neoergasilus japonicus* (Harada, 1930) (Copepoda: Ergasilidae) in Lake Grand Laoucien, France: A Field Study on Life Cycle Parameters and Reasons for Unusual High Population Density

**DOI:** 10.3390/life11101100

**Published:** 2021-10-16

**Authors:** Victor Alekseev, Corinne Cuoc, Dominique Jamet, Jean-Louis Jamet, Remi Chappaz

**Affiliations:** 1 Zoological Institute, Russian Academy of Sciences, Universitetskaya Nab, 1, 199034 St. Petersburg, Russia; 2 INRAE, Aix Marseille University, UMR RECOVER, Case 36, 3 Place Victor Hugo, CEDEX 3, 13331 Marseille, France; remi.chappaz@univ-amu.fr; 3 Mediterranean Institute of Oceanology, Université de Toulon, AMU-UTLN UM110, CS 60584, CEDEX 9, 83041 Toulon, France; jean-louis.jamet@univ-tln.fr (J.-L.J.); d.jamet@univ-tln.fr (D.J.)

**Keywords:** high population density, life cycle, productivity, local factors, energetic flow, microphytobenthos, food relations, host–parasite interactions, near-shore environment, lake volume fluctuation

## Abstract

The fish parasite, *Neoergasilus japonicus* (Harada, 1930), native to Eastern Siberia and the Amur River catchment area, invaded European water bodies in the middle of the last century, possibly due to the human-mediated distribution of fish in the Amur complex (i.e., the genera *Hypophthalmichthys* and *Ctenopharyngodon*). In the deep karst lake, Grand Laoucien (Marseille area, France), this species had an unusually high population density (from 1000 ind./ m^3^ in zooplankton to 4000 ind./ m^3^ in the nearshore area) during the free-living period of its life cycle. The annual cycle of *N. japonicus* includes a 5-month overwintering of fertilized females attached to fish fins and, following this, a five- to six-generation chain from March to November, when the free-living stages in the population alternate with parasite females which attach to their hosts for breeding. The population density of the parasites in zooplankton increased exponentially from spring to autumn, which positively correlated with temperature. We found a strong correlation between *N. japonicus* density and the community development of microphytobenthos, but not between *N. japonicus* and phyto- or zooplankton dynamics. The local contributing factors included a seasonal three-fold decrease in water levels and the development of anoxia in profundal waters, which led to a high ambient fish density and thus susceptibility to the parasite. Although the free-living parasite represented only 1% of zooplankton production, it consumed up to 25% of small invertebrate productivity. The maximum intensity of infection reached 140 parasites per fish, or 4.14 per g of weight. The high infection of fish with this parasite, in our opinion, indicated the danger it poses to the local ichthyofauna, which first encountered this new parasite.

## 1. Introduction

*Neoergasilus japonicus*, a fish parasitic copepod widely distributed in East Asia, was first described in Taiwan [1]. In the late 1960s, it was found in Slovakia and Hungary [2,3], possibly as a result of the introduction of oriental fish. This was followed by findings of the parasite in France, Great Britain, Cuba, Finland and the USA [4,5,6,7,8,9,10]. Since the beginning of this century, it has been recorded in Germany, Italy, Turkey, Mexico, European Russia, Canada and the Czech Republic [11,12,13,14,15,16]. Thus, the species is actively spreading around the world, finding new hosts and changing the existing population relationships.

All the instars in this species, along with males, are free-living organisms. The fertilized female attaches to the fish fins, using their host for feeding and egg producing, as well as overwintering. However, unlike typical ergasilids, unfertilized females of *N. japonicus* can also be found in plankton and can change their hosts. This parasite favors dorsal fins and anal fins are the second preference when infestation increases [10,13,16]. Such a life cycle is accompanied by relatively small modifications of morphology compared to other parasitic copepods [17,18]. Most parasite copepods with a similar life cycle have been partially studied, especially their free-living phases. The density of their populations appears to be low, and the population dynamics in parasitic crustaceans has usually been studied only in females attached to their hosts [19,20,21]. A small number of parasitic copepods were cultivated in the laboratory and they were rarely recorded in nature in sufficient enough numbers to trace their population dynamics by classical hydrobiological methods. Very little is known about where most species live in the water body and which ecological niches they occupy during the free-living period of their life. Food preference and their position in the food web have not been studied in immature ergasilids either [5]. This is especially true in the case of a biological invasion, when a parasite invades a new environment and new hosts and is implemented in new ecological system.

The problems with tracing actual population dynamics in fish parasitic copepods resulted in a lack of knowledge about their role in the energetic budget of the ecosystem they live in. Even though the growth and metamorphosis in *N. japonicus* were studied by Urawa at al. [18], practically nothing is known about its productivity and population dynamics during its free-living period of life.

In the zooplankton of a karstic lake in the Marseille area, we found a natural population of *N. japonicus* with a maximal density of circa 4000 ind./m^3^ in the nearshore area; this was a hundred times greater than the level of ergasilids found in other lakes. This gave us a unique chance to perform a detailed study of the life cycle, productivity and population dynamics of this species. Thus, the purpose of this work was to study the biology of the parasitic invader species in Lake Grand Laoucien, in which the abundance of its free-living phase reached an unusually high density, which made it possible to apply traditional hydrobiological methods and to study the parameters of its life cycle in vivo. An additional goal of this study was to understand the reasons why the density of the *N. japonicus* in this lake was so high. The study also tested the hypothesis that the hydrological features of this karst lake were the main reason for such a high density of the parasitic crustacean population.

## 2. Materials and Methods

Observations were conducted at the small karstic lake, Grand Laoucien (43° N 19′ 862″ 06° E 00′587″, altitude 310 m), during three seasons from 2001 to 2002.

From February 2001 to January 2002, samples of zooplankton, phytoplankton and water were taken, while surface temperature and depth were measured. Sampling and measurements of abiotic parameters were carried out with a frequency of 1–3 times a month. From September 2001 to October 2002 we also collected littoral zooplankton, microphytobenthos, fish and measured the temperature and oxygen profiles with a frequency of 1–2 times every 3–4 months.

The depth of the lake was measured during the period of the maximum level (in spring) using a sounding line, and the changes in this level were assessed each time zooplankton samples were taken using a measuring tape (with an accuracy of 1 cm) and marks on the steep coast section. Chemical analysis of water samples was carried out in the laboratory on the day of sampling.

We sampled surface phytoplankton in 1.5 L plastic bottles and collected benthic microalgae from littoral stones (0.5 m depth) manually using a syringe-like device opening in the anterior part, thus presenting a 3 cm diameter cylinder provided with a scratch plate. In this way, microalgae from an area of about 25 cm² could be pumped into the device. Phytoplankton and microphytobenthos were identified at the species level and counted according to Utermohl [22]. To calculate the algae biomass, a length/volume relationship described by Lohman [23] was used.

Zooplankton samples were taken with a plankton net (diameter 33 cm, mesh size 80 μm) in a place with practically vertical walls and a depth of 5–28 m by filtering 1–3 m^3^ in several close-to vertical hauls. Littoral zooplankton was collected by filtering 50–100 L via the handle net with a mesh size of 80 μm in the near shore area with a depth of 0.5–1.0 m. All samples were preserved in 4% formalin solution. We identified all organisms at the species level and measured their length with an ocular micrometer of 14 μm resolution. For biomasses calculations, we used length/wet weight conversions provided in the literature (Table 1): W (mg) = a * L (mm)^b^. For feeding and productivity rates, we used a method based on the calculation of standard metabolic rate via the animal’s weight and temperature [24]. Similar methods were used for *N. japonicus* but the most important parameters in the species growth were obtained from field sample analyses (see below).

In *N. japonicus* all development stages were separately identified and counted in the zooplankton and littoral samples. In autumn 2001, and winter and summer 2002 we also collected females of *N. japonicus* from fish fins for a host invasion assessment. We separated two types of females on fish fins to note the overlapping among generations. Newly arrived females usually had gray pigmented bodies. Females of the previous generation or older ones had transparent bodies and large egg sacs. These two kinds of females also differed in cepalosome length/width ratios, which were measured under a microscope in formalin-preserved animals. Weight in *N. japonicus* was calculated as in cyclopids (see Table 1).

### 2.1. Life History Parameters

We selected four seasons based on the combination of day length (calendar factor) and actual temperature observed in lake:

Winter: temperature < 5 °C; day length < 12 h per day; duration, 100 days.

Spring: temperature 5–15 °C; day length >12 h per day; 80 days.

Summer: temperature >15 °C; day length >12 h per day; 105 days.

Autumn: temperature 15–5 °C; day length <12 h per day; 80 days.

These seasons were also used for the calculation of the biotic balance in zooplankton and correlative analyses of population dynamics in *N. japonicus.*

Productivity in *N. japonicus* during the free period of life was defined as:

Ps = Cvs* Bs* Ts

where Ts is the duration of a season (spring 60 days, summer 105 days and autumn 60 days), Bs is the average seasonal biomass of *N. japonicus* estimated separately for spring, summer and autumn, and Cvs is the daily production rate for a season.

Data from preserved samples of field population *N. japonicus* were employed to estimate the production rate:

Cv = (ln Wa − ln Wn)/ 4/5GT

where Wa is the weight of adults, (taken from sample analyses); Wn is the weight of the first nauplii, (taken from sample analyses); GT is the generation time (from egg to egg) obtained from parasite population dynamics as an average distance between the two nearest peaks in zooplankton density, adjusted for average temperature of each season (also taken from field observation); and the 4/5 ratio is the part of the generation time that *N. japonicus* spent in plankton as free-living animals; in the experimental study, about 1/5 of the generation time was occupied by settling on fish, the production of eggs and embryonic development [28].

Feeding rate (FR, cal/day) was calculated from:

FR = 1/U*(Pd + R)* Q_10_

where U is food assimilation, 0.7 for mixing diet in *N. japonicus*, R is daily metabolic rate, Pd is the daily production rate, Q_10_ is the temperature coefficient.

Metabolic rate was estimated as a function of body mass (W, wet weight, mg) and expressed as:

R = aW^b^

where a and b are constants. For *N. japonicus*, we used a and b as for other copepods [29].

Daily production was calculated from:

P = Cvs*B

where B is the *N. japonicus* biomass in a sample.

We did not calculate egg production in *N. japonicus* as it was dependent on fish tissue feeding [30].

### 2.2. Diet Evaluation

The ecological role of an organism is largely determined by its position and significance in the food web. For food relations with other hydrobionts, we examined stomach contents in copepodids 3–5 of *N. japonicus* from field samples collected during summer–autumn 2001. In a sample, we chose up to 10 copepodids at random and then dissected their abdomen using fine needles and extracted the digestive tract. Every tract was opened in a drop of glycerol, covered with a cover glass, and observed under a compound microscope under high resolution. We calculated the percentage of empty stomachs and stomachs with algae, as well as the remaining invertebrates, which were identified at the genus level when possible.

### 2.3. Host Sampling

Fish were captured with gill nets of different mesh sizes and placed into the lake in several locations and at several depths. In every fish, we measured weight, length, collected scales for aging and calculated, in the live specimens, the number of parasites attached to the fins, gills and nasal operculum separately.

### 2.4. Statistics

All the data analyzed were first checked for normality and then, appropriate parametric or nonparametric methods from the STATISTICA program were applied. For correlation analyses in *N. japonicus* we used Spearman’s nonparametric correlations. The Mann–Whitney nonparametric test was also used to check two hypotheses: whether 10 °C played the role of a threshold in *N. japonicus* dynamics; and whether the time of microphythobenthos’ presence (July–October) was important for *N. japonicus* dynamics.

## 3. Results

### 3.1. Abiotic and Biotic Parameters of Lake

**Figure 1:**
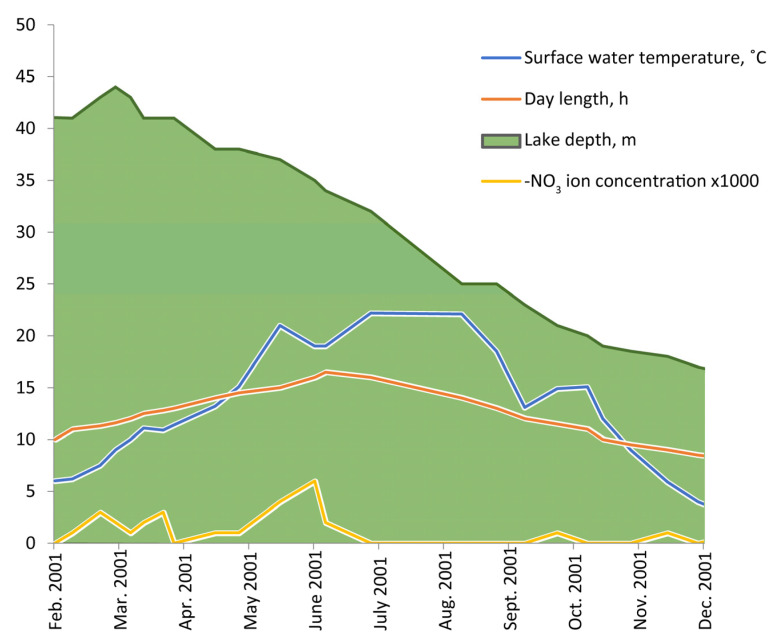
Abiotic parameters evolution in Lake Grand Laoucien in 2001.

The lake level gradually decreased from spring to winter (Figure 1). The maximal depth (45 m) was in March during the rainy season. In 11 months, the lake level decreased to the minimal depth of 14 m in winter. The average rate of depth decrease was about 3 m/month. The water volume in the lake from March to October decreased approximately three-fold.

The vertical temperature and oxygen profiles were analyzed in three typical periods. In January, we observed an isothermal water mass with a high oxygen saturation in the profundal zone. In June, the temperature stratification and the beginning of oxygen limitation in water near the bottom of the lake was discovered. In October, we found a strong temperature stratification in the water column, which was nearly anoxic below 11 m in depth. Inhabitants of the fish zone (above 3 mg O/L) were limited in autumn to the first 6–7 m. Together with the depth evolution, this resulted in an increase in the actual fish population density per cubic meter by at least 9–10 times in comparison with the early spring period.

#### 3.1.1. Phytoplankton and Microphytobenthos Dynamics

**Figure 2:**
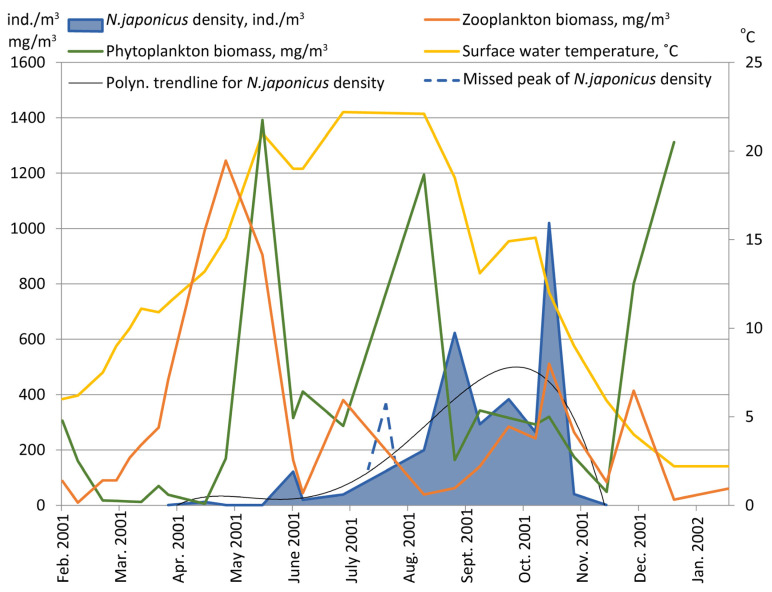
Seasonal dynamics of phyto- and zooplankton biomasses and *Neoergasilus japonicus* population density in Lake Grand Laoucien, 2001.

We identified 47 species of microalgae in both communities. Plankton was dominated by cryptomonads and diatoms most of the time, with *Cryptomonas caudatum* and *Rhodomonas minuta* being the most common species in winter. *Cryptomonas caudatum* and *Ceratium hirundinella* were often found in spring and *Nitzschia closterium*, *Synedra acus* and *S. pulchella* were abundant in the summer months. Plankton algae had several peaks of biomass, with the maximum being at the beginning of summer, when large-sized *Ceratium* cells dominated (Figure 2).

In the summer (approximately from June), microphythobenthos started growing, and in September 2001, all of the stones near the shore were covered with a 3–5 mm deep carpet of the benthic colonial algae, cyanobacteria. This biotope was also inhabited by many invertebrates such as nematodes, chironomids and larvae of other heterotopic insects, ostracods, harpacticoids, eucyclopids and *N. japonicus*. In the autumn the most abundant fish captured by gill nets close to the shoreline were roach.

Based on our observations, in late autumn when temperature decreased to 10 °C, the community of microphythobenthos disappeared very quickly.

#### 3.1.2. Zooplankton Dynamics and Species Composition

We found a very simple zooplankton community in Lake G. Laoucien. One free-living copepod (*Diaptomus vulgaris*), three cladoceran species (*Daphnia cucullata, Ceriodaphnia pulchella, Bosmina longirostris*) and a few rotifers (*Keratella quadrata frenzeli; K. cochlearis macracantha, Filinia major, Polyarthra* spp.) dominated in the lake most of the time. In littoral samples, several cyclopids (*Eucyclops macruroides*, *Paracyclops fimbriatus*), chydorids (*Chydorus sphaericus aleksandrovi*) and littoral rotifers (gen. *Trichocerca, Testudinella, Euchlanis*) were found. The full list of zooplankton species consisted of nine Rotifera, eleven Cladocera and eight Copepoda species, including the poecilostomatoid copepod, *N. japonicus.* All cyclopids belonged to littoral species of the subfamily Eucyclopinae, with *Macrocyclops albidus* being the most common.

In summer, *Daphnia cucullata* was replaced by *Diaphanosoma brachyurum*. As a unique characteristic of the lake, the absence of a true planktonic invertebrate predator must be mentioned. The facultative predator, *Asplanchna priodonta*, was found twice in spring, and could not actually control any species’ population dynamics because its population density was too low (10–60 ind./m^3^).

### 3.2. Population Structure and Dynamics in Free Living Stages N. japonicus

The first copepodids of *N. japonicus* at a density of 12 ind./m^3^ were found in zooplankton in April (Table 2). During summer, the population density of *N. japonicus* had four peaks following one another, with about one month periodicity. This dynamic could apply to four successive generations and resulted in an approximately 100-fold increase in the density of the free-living part of the population.

Nauplii of *N. japonicus* were first found in plankton in late August, and then produced two peaks in early September and early October, separated by a clear depression in density in mid-September.

In early November, almost all copepodites of *N. japonicus* had disappeared from plankton.

Males were practically found in plankton during the whole summer and were never found attached to fishes. Unfertilized females were sampled only three times, with the maximum being at the end of season, and their number ranged from 1 to 7% of the total population density. When males and females were found in plankton together, the sex ratio was 1.5:2 and the males dominated.

### 3.3. Food Composition in N. japonicus

In the gut of *N. japonicus* from June to October, we found four main components: cells of algae (Diatomea and Cyanobacteria dominated all the time), small rotifers (*Trichocerca*, *Brachionus, Polyarthra*), remains of Ciliates and larvae of littoral cyclopids (*Eucyclops* and *Paracyclops*) (Table 3). Practically all these organisms were not abundant in plankton during the summer. Species of Cyanobacteria dominated in microphytobenthos in 2001 and 2002 from June to October but were never found in plankton samples. The larvae of *Eucyclops* were the most abundant in the littoral samples too.

The proportion of empty stomachs varied throughout the year in *N. japonicus* copepodids (see Table 3). In June and in September, animals with empty guts were relatively rare and did not exceed 10% of all copepodids. In October, they become more abundant, and by the end of the month reached 60% of the population. Practically all of those animals were copepodid 5 and, under a histology study, their guts looked similar to those in the animals that were starved. The temperature was relatively stable during October and exceeded 15 °C.

### 3.4. Behavior and Density N. japonicus in Near Shore Area

On 26 September 2001, we tested a hypothesis on the role of water movement in the activation of parasite females to attach to their host. Two samples of 50 L each were collected at the same place in the littoral area (0.3–0.5 m depth) at short intervals. The first sample was collected with minimal water disturbance and did not contain parasites.

The second sample was collected after prior paddle rowing in the water. The important point was that the water movement did not disturb the sediments in the near shore area. This sample contained 19 adults and major copepodites of *N. japonicus*, or about 3800 ind./m^3^. The difference in sampling clearly indicated the important role of fish movement in parasite attraction. The presence of fish in the near shore area in October was confirmed both by visual observation and by gill net catches. This result also showed that the maximal parasite density in autumn was in the littoral zone.

It should be mentioned that the experiment reproduced two extremes that were not produced during usual sampling, in which there was always a certain moderate disturbance of water of approximately the same intensity.

### 3.5. Biomasses and Energetic Flow in Zooplankton

The maximum zooplankton biomass (1.2 g/m^3^) was in spring–early summer, when *D. cucullata* dominated (Figure 2). From the middle of June, the biomass never exceeded 0.5 g/m^3^, and the fast moving (*Diaphanosoma, Diaptomus*) or occasionally littoral species (eucyclopids) produced the bulk of the biomass during late summer and autumn.

Productivity and feeding rates in the zooplankton followed the biomass dynamics due to the minor role of small-sized species such as rotifers and the moderate temperature in summer (Table 4).

Rotifers played a relatively important role in the productivity of the community in wintertime and in spring. Meanwhile, the bulk of annual zooplankton productivity belonged to cladocerans and *Diaptomus*, with the maximum productivity was observed in the spring–summer period.

Predators such as adult cyclopids produced about 2% of the total zooplankton production, and *N. japonicus* contributed about 0.4% into the energetic flow, or 25% of the predator consumption. The food consumption in predators was about 1000 cal/m^3^ per year, with the maximum consumption occurring in the summer. In the summer, the amount of food consumed by *N. japonicus* was as much as the total rotifer and littoral eucyclopid nauplia production. In autumn, the food consumption by *N. japonicus* was about 10 times higher than the rotifer production. All of these animals, such as cyclopid nauplia, and rotifers, etc., were found in the digestive tract of the parasite, and their populations could be controlled by *N. japonicus* predation, especially at the end of the season and in the near shore area.

The annual production of zooplankton was about 5713 cal/m^3^ per year, which fitted to the mesotrophic lake level. The zooplankton community in the lake had a positive balance of energetic flow (about 4800 cal/m^3^ per year), with the maximum production in early summertime. The late summer and autumn zooplankton production was three and six times lower, respectively, than at the beginning of season.

### 3.6. Fish

All four fish species known in Grand Laoucien were captured by coastal gill nets in 2001–2002 (Table 5).

The dominative species was roach, *Rutilus rutilus* (L., 1758), presented in all four catches. In October 2001 and January 2002, the average sizes of the individuals captured ranged from 12 to 18 cm, which corresponded to 2–3-year-old individuals (from 2+ to 3+). In June and July 2002, the specimens that were captured ranged from 11.5 to 13 cm in size (2+).

The second place was taken by chub, *Leuciscus cephalus* (L., 1758) which was found three times. The dominant age class was formed by individuals of 12–15 cm in size (2+); however, in July 2002, a few small individuals were captured with sizes of 7–8 cm (1+).

Two Perciformes species, the Eurasian perch *Perca fluviatilis* (L., 1758) (16–18 cm, 2+/ 3+) and the alien species from America, the pumpkinseed sunfish *Lepomis gibbosus* (L., 1758), were collected only once per year, in summer/autumn, respectively.

Although the roach was the leader in the number of infected fish, the invasive *L. gibbosus* demonstrated the highest infection intensity if calculated per weight of fish (see Table 5). Most frequently, parasites were attached to the dorsal and anal fins of the fish.

### 3.7. Correlative Analyses of N. japonicus Density with Environmental Cues

We applied Spearman’s nonparametric correlation to analyze *N. japonicus* density and some variables regarding the environment, such as water level, ion (NO_3_,SO_4_,HCO_3_,Cl, Ca, Mg, Na, K) concentration, phytoplankton and zooplankton biomass, temperature, and day length. The correlations with a P-level of less than 0.05 are mainly discussed here.

Weak but significant correlations were found between the parasite density and the adult cyclopids and cladoceran biomass. Significant moderate negative correlations also existed between *N. japonicus* and the rotifer (possible prey) biomass.

A positive correlation was found between *N. japonicus* density and such environmental cues as temperature and day length. The obtained correlations were used to formulate and test two hypotheses:

(1) whether 10 °C plays the role of a threshold in *N. japonicus* dynamics;
(2) whether the time of microphythobenthos’ presence (July–October) is important for *N. japonicus* dynamics.

Both hypotheses were confirmed. We found that the period of time with a temperature above 10 °C was strongly related to the population dynamics of *N. japonicus* (Z = −3.818; *p* < 0.001). Other temperatures tested showed a lower level of correlation (7, 15 °C ) than 10 °C or were not confirmed by the Mann–Whitney test (20 °C ).

A relationship between the time of the microphytobenthos’ development and population density in *N. japonicus* was also confirmed (Z = −3.910; *p* < 0.001). This could be explained by the strong correlation between the temperature and microphytobenthos’ dynamics that was also found.

## 4. Discussion

The small (diameter 150 m) but deep (45 m) karstic Lake Grand Laoucien has practically vertical walls. From June, its surface is covered with dense attached algal colonies (microphytobenthos) that become a dominant algal community in the autumn. It is a permanent, monomictic, Mediterranean-type lake with a water level fluctuating markedly throughout the year.

Seasonal changes in water chemistry parameters did not follow the depth evolution that could be seen on the NO_3_ ion evolution (see Figure 1). This confirmed the conclusion of Kilian [31] that the main reason for the water level changes in this lake was not evaporation but filtration into ground water, otherwise, one would observe a gradual increase in the chloride/sulphate concentration of the water. In our observations, even in December when the lake depth was minimal, after temperature mixing, the concentrations of the main ions in the water were at the same level or even less than in spring.

The annual surface water temperature correlated with day length. The temperatures which were important for turnover processes (less than 10 °C) were observed at the end of March and at the end of October. From the middle of May to the middle of October, the temperature of the surface water in the lake was above 15 °C, and so it was favorable for most of the invertebrate species found in the lake.

The lake has four fish species and a very simple plankton community.

The roach (*Rutilus rutilus*) is a typical benthophage feeding on both plant and animal food, which it can collect from the lakebed and from rocky surfaces, including thickets of higher aquatic vegetation. Quite often, especially in the second half of the summer, algae are found in its diet, which grow en masse in the coastal zone of lakes. The classic habitat for the small roach is thickets of higher aquatic vegetation, while the large roach moves to deeper areas during the daytime; with the onset of dusk it also comes to the shore to feed. The roach spawns in the spring and lays eggs in the higher vegetation, and this is also one of the reasons why it comes close to the shore. The chub (*Leuciscus cephalus*) is a planktonic species, with an upper mouth designed to collect food from the surface of the water, from which it often collects fallen insects. With a shortage of such food, it can feed in the littoral zone of lakes, where its main food is the larvae of aquatic insects; the species also spawns near the coast. The European perch (*Perca fluviatilis*) is a predatory fish, capable of feeding on other small fish, but small perches eat aquatic insects and keep to the coastal water area. Their schooling behavior is very characteristic, including joint hunting for fry (young fish of the current year); in the reservoir, they usually keep to pits or underwater shelters, fallen trees, and large stones, etc. The shores of Lake Grand Laoucien, paved with slabs of limestone, provide many such shelters. The pumpkinseed sunfish (*Lepomis gibbosus*) is a North American fish, introduced in the 19th century to the reservoirs of Europe as an object of sport fishing and was actively spread by fishing societies, including in isolated reservoirs. It is quite possible that it was introduced into Lake Grand Laoucien, which has no connection with other water bodies, in a similar way.

The intensity of infection was recorded from 1–2 ind. per host (*Rutilus rutilus* and *Perca fluviatilis*) to 50–60 ind. per host (*Leuciscus cephalus*) and 145 ind. per host (*Lepomis gibbosus*). Most of the European findings demonstrate: 1–2 ind. per host [11,14] or 7–50 ind. per host [8,13]. The study of Ondrackova et al. [16] showed a high infection intensity (up to 99 ind. per host) of the alien species from North America, *Lepomis gibbosus*, with *Neoergasilus japonicus*. Apparently, it is no coincidence that this particular species in Lake Grand Laoucien had the highest level of infection intensity.

### 4.1. Description of Life Cycle in N. japonicus

The biological peculiarities of *N. japonicus* in experimental conditions were studied by Urawa et al. [28]. In many respects our field observation fitted to their experimental data (Table 6). Such important biological features as fecundity and generation time in *N. japonicus* populations from the Hiroshima and Marseille areas were practically equal. The observed differences in the durations of overwintering and active phases of the parasite life cycle in Japan and France, in our opinion, at most depended on the latitudinal characteristics of the places where the two populations lived.

Fertilized but egg-free *N. japonicus* females spent the cold season attached to fish fins, at least from November to February. They had sperms in *receptaculum seminis* so their eggs could be laid later [30]. No free-living stages were found in plankton in Lake G. Laoucien between November 2001 and April 2002, highlighting the fact that free-living immature stages of this parasite could not survive the winter without the fish. The effectiveness of surviving among overwintering females in this lake could be estimated at almost 100%, since no significant difference was found in the parasite infestation of roach collected in October and January. For other poecilostomatoid parasites, such as *Ergasilus sieboldin* (Nordmann, 1832) from Northern Russia, a much less effectiveness of overwintering (50–85%) was observed [32]. This was possibly caused by the longer winter period in Russia than in the Mediterranean area.

In spring, *N. japonicus* in Lake G. Laoucien started breeding. The exact time of this was not determined directly nor by observation of the fish, as they were not captured in spring or in plankton samples, possibly because nauplii had a low density and/or low level of survival at this time. At first, *N. japonicus* copepodids with a density of 12 ind./m^3^ were found on 30 April. To our mind, this was the onset of the breeding in the overwintering females. We calculated the approximate time of the beginning of breeding as late March on the basis of the duration of the generation time (see below).

The second time we found *N. japonicus* in zooplankton as copepodids 2–5 and males was on 15 June. The 45-day interval between these two peaks should be regarded as exceeding the generation time because, on 30 April, we found only copepodid 3, while on 15 June, at least part of the population of *N. japonicus* had become adults. Urawa et al. [28] supposed that, in *N. japonicus*, nauplii and copepodid larvae showed isochronal development. As copepodid 3 is the ninth of the 12 development stages known in the species, we can thus calculate the second generation time as about one fourth shorter than what we attained from the distance between the two peaks in April and June. This provides a generation time in spring of 34 days under an average temperature for this period of 17 °C, or 28.9 days if converted to 20 °C.

The next time *N. japonicus* was found in a sample was on 20 August, but there was no doubt that we missed one generation in between because of the 40-day gap in sampling from 11 July. To calculate the time of the two following generations together, we proposed that the sharp peak in parasite density on 7 September (more than 600 ind./m^3^) was the greatly reduced remains of the fourth generation. The high proportion of naupliar stages at this time (about 50% of the population density) also indicated this date as a new generation peak. After a simple calculation, we found the generation time to be 28.5 days for the summer period with an average temperature of 21 °C, or 29.9 days if converted to 20 °C.

More accurately, we can estimate the time between the generation of *N. japonicus* in autumn from the two peaks of density on 7 September and 5 October which coincided with sharp increases in naupliar density. This gives a 28-day duration for the generation time in *N. japonicus* under an average temperature of 17 °C, or 24 days if converted to 20 °C which fits very well to the experimental observations on the ontogenetic development in this species [28].

The last generation could be identified with the maximum density at the end of October, or by the date of practical absence from the plankton on 8 November. This lasted 21–32 days under an average temperature of 15–13 °C, respectively, or 16–21 days if converted to 20 °C. This duration of generation time was obviously shorter than what we found in the summer generation. This could be attributed to special features of overwintering females. We did not use the latter data for the calculation of the average generation time for *N. japonicus*.

Now we could calculate the number of generations in *N. japonicus* from its first appearance (30.04) to the disappearance of the free-living stages in the community (30.10). As the average temperature for the 6-month active growing period in *N. japonicus* was also 17.1 °C, we had approximately 3000 degree days which could have resulted in five generations of *N. japonicus* that lasted 27.6 days under 20 °C, or 535 degree days each. One more generation should be added to this number as we started our study from the overwintering females. This calculation provides an additional confirmation that in the July–August period, where we had a gap in sampling, there were two succeeding generations.

If the calculated duration of the development time for copepodid 3 was ¾ of the generation time or 400 degree days, then we must add the egg development time (60 degree days according to Urawa et al., [28]) and compare it with the retrospective temperature from 30 April to early spring time. One could find that the start of the first generation would be at the end of March, when the water temperature first exceeded 10 °C. The same temperature threshold was found for the end of the population in the free-living stages of *N. japonicus* in November. This could mean that the active period of the life cycle in *N. japonicus* was limited by temperatures above 10 °C, and the period between these spring and autumn temperature borders presented an optimal time for this parasite’s development and should be correlated with population density in *N. japonicus*. This hypothesis was tested and strongly confirmed by the Mann–Whitney test (Z = −3.818; *p* < 0.001).

### 4.2. Food Relations and the Role of N. japonicus in Zooplankton Energetic Budget

The copepodid stages of *N. japonicus* in our lake were facultative predators of small animals such as protists, rotifers and nauplii of other copepods. In the stomachs of the copepods, cells of Diatomea and Cyanobacteria were also found. Therefore, ergasilids combined predation with algal consumption, as many other cyclopids did [33].

A possible difference in diet between *N. japonicus* and cyclopids seems to be based on the construction of mouth appendages in the copepods. Cyclopids use their maxillae and maxilipeds to grasp and hold prey, while the mandibles break up and grind it. That is why cyclopids can consume relatively large organisms, sometimes even larger than the predator itself [33].

In *N. japonicus*, the second antenna is transformed into a claw for host attachment, while other mouth appendages are reduced. The lack of maxillipeds and the reduction in maxillules and mandibles in the mouth complex of *N. japonicus* does not favor grasping, holding and destroying large-sized prey. We never found parts of other crustaceans in the stomach of ergasilids, but only complete bodies of larvae of cyclopids and ostracods. The maximal size of prey consumed by *N. japonicus* seemed to be limited by the mouth opening diameter. We found that the largest prey was a young ostracod, with a length of about 300 μm, and nauplii and early copepodids of *Eucyclops*, also about 300–400 μm in length. All these animals were not crushed.

**Figure 3:**
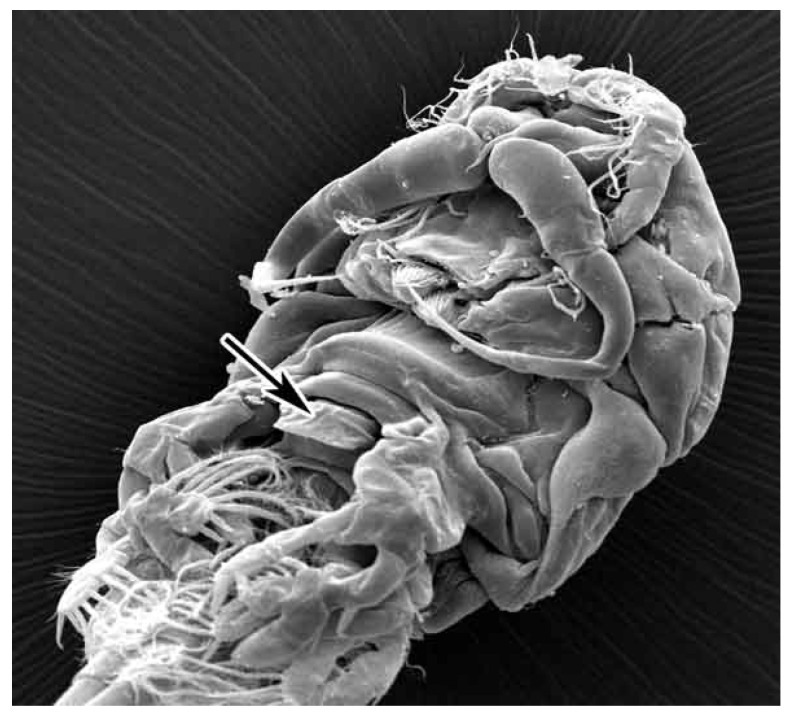
Prosome of *Neoergasilus japonicus*, ventral. Lake Grand Laoucien, France.

Among the most common food components in *N. japonicus* stomachs, were colonies of Cyanobacteria and diatoms that could be scraped off stones or other hard substrates. It is possible that the spade-like armament between the cephalosome and urosome with undefined functions, indicated with an arrow in Figure 3 is used by *N. japonicus* to scrape off the sedentary algae species from a hard substrate.

With such limitations for prey grasping and consuming in *N. japonicus*, it can still be an effective hunter if the prey is small enough to swallow, moves slowly, as with rotifers, ostracods or *Eucyclops* nauplii, and is abundant, as with protists when they are at high densities. All of these animals during the summer–autumn period are concentrated in the near shore area and only occasionally appear in plankton.

Phyto- and zooplankton’s biomass dynamics in the lake had maximums at the beginning of summer, when the density of *N. japonicus* was low. In summer, in these communities, relative population depressions were observed. We did not find any significant correlation between *N. japonicus* density and phyto- or zooplankton biomasses.

In rotifers and small sized crustaceans, the common prey of *N. japonicus*, peaks of biomass were observed in April when the first generation of *N. japonicus* grew. There is no doubt that *N. japonicus* could not be the main reason for the rotifer density decline in April, as food consumption in this parasite population was many times less than the productivity rate of rotifers in spring. Cladoceran competition was a widely known factor causing rotifer decline during this period of seasonal succession in lakes.

In summer and especially in autumn, the food consumption in the *N. japonicus* population increased significantly, and we found a strong negative correlation between rotifer biomass/productivity and the density of *N. japonicus*.

Regarding the food composition in ergasilids in summertime, we can conclude that they found food in the microphytobenthic community, where algae species, protists and eucyclopid nauplii were abundant. This was confirmed by a strong positive correlation between *N. japonicus* density and the time of microphytobenthos’ abundance in the lake. The experiment examining the response of the parasite to water movement in the near shore area also confirmed this conclusion.

Even though the role of *N. japonicus* in seasonal zooplankton productivity was minor and did not exceed 0.4% of the total energetic balance of the community, the impact of parasite predation on small-sized animals in autumn was important. In autumn, when the population density of *N. japonicus* was maximal, parasite predation reached up to 50% of predator grazing in the near shore area. The impact of *N. japonicus* in zooplankton grazing was enough to control rotifers and cyclopid nauplii and could be the reason for their depression in autumn, especially in the near shore area.

### 4.3. Reasons for Such a High Density of N. japonicus in the Lake

Important conditions which have contributed to population success in fish parasites include:

(1) A decrease in fish habitat, and thus a higher fish density due to seasonal lower water levels and the development of hypolimnetic hypoxia;
(2) The importance of nearshore benthic algae for fish foraging;
(3) A simple zooplankton community in which invertebrate predators are absent.

The first factor depends on hydrological conditions found in the lake caused by the dramatic changes in the water level and lake temperature stratification in June, which led to an exponential increase in fish density. This increase in fish density must be an important factor for the high effectiveness of parasite settlement on their hosts in the summer and autumn.

The second factor has a biological origin. The decline in the phyto- and zooplankton productivity, as well as the death of benthos due to anoxia, also began around June, but this was followed by a jump in biomass in the microphythobenthos community in the littoral zone. In lakes, the productivity of benthic algae mainly depends on light penetration into the water column and is limited to a two-Secci disk depth of about 3.5–4 m in the lake during summer. In the karstic lake with practically vertical walls, this photosynthetic area was formed as a very narrow belt of sedentary algae close to the shoreline. Fish foraged in this area, as it was the only place where they could find food. As free-living stages of *N. japonicus* grew in this near shore community, they could easily come into contact with fish when female parasites were ready to attach onto hosts.

Finally, the very simple zooplankton structure with invertebrate predators missing in Grand Laoucien could be also important for parasite survival. Kasahara [34] observed a biological control of nauplia *L. cyprinidacea* by cyclopid predation. A similar effect is quite possible for *N. japonicus* too, as its nauplii are the appropriate size to be eaten by other cyclopid species. In Grand Laoucien in 2001, populations of free-living cyclopids as well as many other invertebrate predators were too low to control *N. japonicus*.

All these coincidences worked in the same way, which, in our opinion, resulted in the progressive growth of parasite density and multiplication with every new generation. This conclusion was supported by the exponential population growth found in *N. japonicus* in 2001, when the density of the population increased more than 100-fold within 6 months.

The most likely route for this parasite to enter this isolated lake is by human-mediated dispersal, since this reservoir was used for fishing competitions and, for this purpose, was inhabited by species of which were important for this sport, including *L. gibbosus*, which showed the highest infection intensity among all species, which most likely indicates the introduction of parasites.

## 5. Conclusions

The invader from the Far East, *N. japonicus*, is known as a parasite of cyprinids which was confirmed in Lake Grand Laoucien, France. The wide distribution of this new parasite in the lakes and rivers of Europe created the appearance of additional negative factor, as well as with an anthropogenic effect on mass fish populations such as roach (*Rutilus rutilus*) and crucian carp (*Carassius carassius*). At the same time, it showed a high plasticity in host selection and was found in a high density in *L.gibbosus*, an invasive Perciformes fish from North America, as a parasite. This fact increases the possible danger represented by the invasion of *N. japonicus* for the European native fish population.

The life cycle and biological features of the parasitic crustacean, *N. japonicus*, in the karstic Lake Grand Laoucien almost completely coincided with that of the studied range in Japan, which indicated a good correspondence with the habitat conditions in the new range for the parasite.

The proposed hypothesis about the leading role of hydrological environmental factors in the karstic Lake Grand Laoucien in the formation of an unusually high population size during the free period of its existence (up to 1000 ind. per m^3^ in zooplankton and 4000 ind. per m^3^ in the nearshore area) was confirmed. Conditions that contributed to the population success in *N. japonicus* include: the significant decrease in water level and volume during the season, strong anoxic conditions in deep water during most of summer–autumn period, the low effect of invertebrate predators, and the domination of peryphyton as the main primary productive community in the lake from June to November. Among the biotic factors favoring the high population density of *N. japonicus*, the population and biomass dynamics of the microphytobenthos community in the littoral zone, where the main population of the parasite was concentrated, were of the greatest importance, as confirmed by correlational analysis. All these factors working together increased the possibility of parasites coming into contact with their hosts (fish) in the near shore area.

The free-living stages of parasitic crustaceans, due to their usually low population density, had never been included in the bioenergy balance of water bodies before. This study, carried out with a uniquely large population of *N. japonicus*, made it possible to evaluate its role in the production and especially in the diet of free-living carnivorous invertebrates. The most significant influence of *N. japonicus* on the planktonic ecosystem was noted at the end of the growing season during the period of its maximal abundance. In late summer and at the end of the season, *N. japonicus* could control rotifers and nauplii of benthic cyclopids as the main prey and consume up to 50% of their population during the free-living period.

## Figures and Tables

**Table 1:** Length/wet weight conversion coefficients for zooplankton used for zooplankton calculation in Lake Grand Laoucien.

Organisms	a	b	Authors
Rotifers			
*Asplanchna*	0.23	3	Ruttner-Kolisko [25]
*Keratella cochlearis*	0.02	3	
*K. quadrata*	0.22	3	
*Polyarthra* sp.	0.38	3	
*Filinia*	0.12	3	
Cladocerans			
*Bosmina*	0.176	2.97	Balushkina and Winberg [26]
*Ceriodaphnia*	0.141	2.77	
Chydoridae	0.203	2.77	
*Daphnia*	0.075	2.93	
*Diaphanosoma*			
Copepodes			
Nauplii all groups	0.054	2.45	Alekseev [27]
*Diaptomus* Cop+Adults	0.037	2.81	
Cyclopides *	0.034	2.92	

* Including Neoergasilus japonicus.

**Table 2:** *Neoergasilus japonicus* population structure, Lake Grand Laoucien, 2001, ind./m^3^.

Dates	Nauplii	C1	C2	C3	C4	C5	Male	Female	Total
30.04.01	0	0	0	12	0	0	0	0	12
11.05.01	0	0	0	0	0	0	0	0	0
30.05.01	0	0	0	0	0	0	0	0	0
15.06.01	0	0	49	24	24	0	24	0	121
20.06.01	0	0	10	5	5	0	0	0	20
11.07.01	0	0	10	20	5	0	5	0	40
22.08.01	17	91	59	13	7	7	7	0	201
07.09.01	279	143	156	26	7	7	7	0	625
20.09.01	34	98	111	26	7	7	0	13	296
05.10.01	206	61	56	22	17	22	0	0	384
19.10.01	17	33	26	39	46	52	33	20	266
26.10.01	0	260	180	160	160	140	80	40	1020
08.11.01	0	0	0	0	0	0	0	0	0

**Table 3:** Digestive gut content in copepodids 3–5 of *Neoergasilus japonicus* (% of copepodids with the component) in Lake Grand Laoucien.

Components	05.06.01	19.09.01	26.10.01
Cyanobacteria	90	80	40
Diatomea	20	10	10
Other algae	10	10	10
Infusoria	80	0	0
Rotatoria	0	40	0
Nauplia Eucyclopids	20	30	20
Other invertebrates	0	10	0
Empty	10	20	60

**Table 4:** Elements of energetic balance in zooplankton/littoral communities and in population *Neoergasilus japonicus*, Lake Grand Laoucien, 2001–2002, productivity (P, cal/m^3^ per season), feeding rates (C, cal/m^3^ per season), biomass (B, mg/m^3^) and annual P/B coefficients (year^−1^).

Rotatoria			
Seasons	B, mg/m^3^	P, cal/m^3^	C, cal/m^3^
Spring	13.3	72.0	289.0
Summer	2.6	42.7	177.0
Autumn	0.4	2.6	10.0
Winter	3.4	37.7	93.4
Annual	4.8	155.0	570.0
P/B per year	53.1		
	Cladocera		
Spring	208.0	507.8	2413.5
Summer	266.3	2512.0	1,1951.0
Autumn	60.1	203.0	941.0
Winter	11.5	34.2	164.5
Annual	278.0	3257.0	15470.0
P/B per year	19.5		
Calanoida+Naup			
Spring	93.6	302.0	2364.0
Summer	137.0	1328.0	10,382.0
Autumn	189.6	380.0	3028.0
Winter	118.0	164.0	1279.0
Annual	140.0	2174.0	17,053.0
P/B per year	25.9		
Cyclopoida			
Spring	0	0	0
Summer	0.6	28.8	120.0
Autumn	38.2	79.2	594.0
Winter	0.1	10.0	64.0
Annual	8.8	118.0	778.0
P/B per year	22.3		
*N. japonicus*			
Spring	0	0.1	1.1
Summer	0.5	7.8	95.8
Autumn	2.6	15.5	121.4
Winter	0		
Annual	0.7	23.4	238.0
P/B per year	56.0		

**Table 5:** Seasonal fish invasion by fish parasite, *Neoergasilus japonicus*, in Lake G. Laoucien in 2001–2002.

Date		*R. rutilus*	*L. cephalus*	*P. fluviatilis*	*L. gibbosus*
10.10.01	number of fish, ind.	**11**	**4**		**1**
	weight of fish, g	**89.4**	**109** **.** **9**		**35.0**
	mean + standard error	1.77	9.64		
	number of *N. japonicus*	**18.4**	**239+**		**145.0**
	mean + standard error	5.67	97.5		
	number of *N. japonicus* per fish weight, ind/g	**0.21**	**2.124**		**4.14**
	mean + standard error	0.066	0.793		
29.01.02	number of fish, ind.	**13**	**2**		
	weight of fish, g	**79.7**	**30.3**		
	mean + standard error	9.08	1.25		
	number of *N. japonicus*	**20.5**	**102+**		
	mean + standard error	7.79	32.63		
	number of *N. japonicus* per fish weight, ind/g	**0.21**	**3.33**		
	mean + standard error	0.074	0.953		
11.06.02	number of fish, ind.	**3**			
	weight of fish, g	**73.1**			
	mean + standard error	3.21			
	number of *N. japonicus*	**3.7**			
	mean + standard error	0.91			
	number of *N. japonicus* per fish weight, ind/g	**0.05**			
	mean + standard error	0.011			
29.07.02	number of fish, ind.	**5**	**2**	**7**	
	weight of fish, g	**64.2**	**8.9**	**122**	
	mean + standard error	6.54	1.2	17.6	
	number of *N. japonicus*	**10.5**	**0.5**	**2.7**	
	mean + standard error	4.4	0.5	0.6	
	number of *N. japonicus* per fish weight, ind/g	**0.18**	**0.06**	**0.01**	
	mean + standard error	0.066	0.065	0.005	

**Table 6:** Life cycle parameters in *Neoergasilus japonicus* in Japan (Hiroshima Pref., 34 °N) and in France (Lake G. Laoucien, 44 °N).

Life Cycle Parameters	France, Field Observation	Japan, Experimental Data
	(present study)	[28]
Fecundity, egg/female	60–68	64–78
Duration overwinter phase, days	130	120
Duration reproductive phase, days	235	245
Duration (20 °C), days:		
Development from egg to adult		21
Mating and setting to host *		3
Egg development		3
Total generation time	27.6 (24–30)	27 **

* Includes the time for first clutch production, ** In Urawa et al. 1991 the generation time was estimated in 24 days as they did not include mating and setting to host time.

## Data Availability

The data that support the findings of this study are available from the author upon reasonable request.

## References

[1] Harada I. (1930). Studies on the freshwater fauna of Formosa (I). A new copepod species parasitic on Formosan freshwater fish. J. Trop. Agric..

[2] Hanek J. (1968). The finding of *Neoergasilus japonicus* (Harada, 1930) (Copepoda: Ergasilidae) in Europe. Folia Parasitol..

[3] Ponyi J., Molnar K. (1969). Studies on the parasite fauna of fish in Hungary. V. Parasitic copepods. Parasitol. Hung..

[4] Lescher-Moutoue F. (1979). Presence en France du copepode Ergasilidae *Neoergasilus japonicus* (Harada). Crustaceana.

[5] Fryer G. (1982). The Parasitic Copepoda and Branchiura of British Freshwater Fishes.

[6] Mugridge R.E.R., Stallybrass H.G., Hollman A. (1982). *Neoergasilus japonicus* (Crustacea: Ergasilidae). A parasitic copepod new to Britain. J. Zool..

[7] Prieto A., Fajer E., Vinjoy M. (1985). *Neoergasilus japonicus* (Copepoda: Ergasilidae) en peces en cultivo intensivo en Cuba. Revista de Salud Animal.

[8] Tuuha H., Valtonen E.T., Taskinen J. (1992). Ergasilid copepods as parasites of perch *Perca fluviatilis* and roach *Rutilus rutilus* in Central Finland—seasonality, maturity and environmental influence. J. Zool..

[9] Hayden K.J., Rogers W.A. (1998). *Neoergasilus japonicus* (Poecilostomatoida: Ergasilidae), a parasitic copepod new to North America. J. Parasitol..

[10] Hudson P.L., Bowen C.A. (2002). First record of *Neoergasilus japonicus* (Poecilostomatoida: Ergasilidae), a parasitic copepod new to the Laurentian Great Lakes. J. Parasitol..

[11] Knopf K., Holker F. (2005). First report of *Philometra obturans* (Nematoda) and *Neoergasilus japonicus* (Copepoda) in Germany. Acta Parasitol..

[12] Alfonso G., Belmonte G. (2010). *Neoergasilus japonicus* (Harada, 1930): A new non-indigenous copepod for the Italian fauna. Ital. J. Zool..

[13] Soylu E., Soylu M.P. (2012). First record of the nonindigenous parasitic copepod *Neoergasilus japonicus* (Harada, 1930) in Turkey. Turk. J. Zool..

[14] Sokolov S.G., Reshetnikov A.N., Protasova E.N., Voropaeva E.L. (2017). New data on alien species of parasites and hosts in the ecosystem of Lake Glubokoe (Moscow oblast, Russia). Russ. J. Biol. Invasions.

[15] Marshall C.C., Hudson P.L., Jackson J.R., Connolly J.K., Watkins J.M., Rudstam L.G. (2019). First record of the non-indigenous parasitic copepod *Neoergasilus japonicus* (Harada, 1930) in the Lake Ontario Watershed: Oneida Lake, New York. J. Great Lakes Res..

[16] Ondrackova M., Fojtu J., Seifertova M., Kvach Y., Jurajda P. (2019). Non-native parasitic copepod *Neoergasilus japonicus* (Harada, 1930) utilizes non-native fish host *Lepomis gibbosus* (L.) in the floodplain of the River Dyje (Danube basin). Parasitol. Res..

[17] Yin W.Y. (1956). Studies on the Ergasilidae (parasitic copepoda) from the freshwater fishes of China. Acta Hydrobiol. Sin..

[18] Urawa S., Muroga K., Kasahara S. (1980). Naupliar development of Neoergasilus japonicus (Copepoda: Ergasilidae). Nippon Suisan Gakkaishi.

[19] Gusev A.V. (1985). Keys to parasites of freshwater fish of the USSR. Parasitic Metazoa.

[20] Nie P., Yao W.J. (2000). Seasonal population dynamics of parasitic copepods, Sinergasilus spp. on farmed fish in China. Aquaculture.

[21] Idoumou M., Nizar S., Mustapha A., Sanaa Y., Khadija El K., Abdechahid L., Belghyti D. (2019). Population Dynamics of Copepods (Lernaea Cyprinacea Linnaeus, 1758) Tilapia Parasites from the Senegal River-Mauritania. Examines Mar. Biol. Oceanogr..

[22] Utermöhl H. (1958). Zur Vervollkommung der quantitativen Phytoplankton-Methodik. Mitt. Int. Ver. Limnol..

[23] Lohmann H. (1908). Untersuchungen zur Feststellung des vollständigen Gehaltes des Meeres an Plankton. Wiss. Meeresunters. Abt. Kiel N. F..

[24] Edmonson W.T., Winberg G.G. (1971). A manual on methods for the assessment of secondary productivity in fresh waters. IBP Handbook.

[25] Ruttner-Kolisko A. (1975). The vertical distribution of plankton rotifers in a small alpine lake with a sharp oxygen depletion (Lunzer Obersee). Verh. Int. Ver. Limnol..

[26] Balushkina E.V., Winberg G.G., Winberg G.G. (1979). Relation between body mass and length in planktonic animals. Obshchiye Osnovy Izucheniya Vodnykh Ekosistem.

[27] Alekseev V.R. (1980). The Growth, Development and Production of Freshwater Cyclopids in Water Bodies of the River Volga Delta. Ph.D. Thesis.

[28] Urawa S., Muroga K., Kasahara S. (1991). Growth and fecundity of the parasitic copepod *Neoergasilus japonicus* (Ergasilidae). Bull. Plankton Soc. Japan.

[29] Sushchenya L.M. (1975). Quantitative Patterns of Nutrition in Crustaceans.

[30] Baud A., Cuoc C., Grey J., Chappaz R., Alekseev V. (2004). Seasonal variability in the gut ultrastructure of a parasitic copepod *Neoergasilus japonicus* (Copepoda, Poecilostomatoida). Can. J. Zool..

[31] Kilian W. (1906). Essai d’une monographie hydrologique des environs de Gareoult (Var). Bull. Serv. Carte Geol. France.

[32] Bauer O.N. (1959). Ecology of fresh water fish parasites. Izvestiya Gosudarstvennogo Nauchno-Issledovatelskogo Instituta Ozernogo i Rechnogo Ribnogo Khozyaistva.

[33] Monakov A.V. (2003). Feeding of Freshwater Invertebrates.

[34] Kasahara S. (1962). Studies on the biology of the parasitic copepod Learnea cyprinacea Linnaeus and the methods for controlling this parasite in fishculture ponds. Contr. Fish. Lab. Fac. Agric. Univ. Tokyo.

